# Design of a quasi-experiment on the effectiveness and cost-effectiveness of using the child-interview intervention during the investigation following a report of child abuse and/or neglect

**DOI:** 10.1186/1471-2458-13-1164

**Published:** 2013-12-11

**Authors:** Froukje Snoeren, Cees Hoefnagels, Francien Lamers-Winkelman, Paul Baeten, Silvia MAA Evers

**Affiliations:** 1 Trimbos Institute, Netherlands Institute of Mental Health and Addiction, P.O. Box 725, 3500 AS, Utrecht, The Netherlands; 2 CAPHRI, School for Public Health and Primary Care, Maastricht, Netherlands; 3 VU University, Amsterdam, The Netherlands; 4 Advies- en Meldpunt Kindermishandeling Haaglanden, The Hague, The Netherlands

**Keywords:** Child maltreatment, (Cost-)effectiveness, Economic evaluation, Quasi-experiment, Advice and reporting center on child abuse and neglect, Child-interview intervention

## Abstract

**Background:**

In the Netherlands, suspected cases of child maltreatment can be reported to an advice and reporting center on child abuse and neglect (Advies- en Meldpunt Kindermishandeling or AMK). AMKs investigate these reports, screen for problems in the family and its surroundings and refer to child welfare. Over the last decades the focus of AMK investigations has changed from an adult-only approach to a more child-oriented approach using a Child-Interview intervention. The effects and cost-effectiveness of AMK involvement in the Netherlands have never been studied. The primary aim of this study is therefore to examine the effect of the participation of maltreated children aged 6–18 years in the Child-Interview intervention on their mental health and quality of life. As a second aim, this study will examine the balance between additional costs and effects of the Child-Interview intervention in comparison with AMK investigation without the Child-Interview intervention (adult-only intervention).

**Design/Methods:**

A quasi-experiment will be performed consisting of two post-intervention measurements of two nonequivalent groups: an intervention group, in which the Child-Interview intervention has been used during the AMK investigation, and a control group, in which the intervention has not been used (adult-only intervention). Participants from an ongoing prospective study on the mental health and quality of life of maltreated children after a report to an AMK, will be contacted to complete a questionnaire twice. Multivariate regression analyses will be used to determine effectiveness of the Child-Interview intervention. The economic evaluation will involve a cost-effectiveness analysis and a cost-utility analysis.

**Results/Discussion:**

This will be the first study to examine the effect of AMK involvement in the Netherlands. Using the Child-Interview intervention during AMK investigation may prevent or reduce negative outcomes of child maltreatment, which may result in a lower consumption of healthcare and other services. In addition, the importance of economic evaluations is increasingly recognized, and economic evaluations about child maltreatment are scarce. Limitations include the risk of potential recall bias and selection bias.

**Trial registration:**

NTR3728, funded by ZonMw, project 15700.2012

## Background

It is commonly known that child maltreatment has a huge impact on the lives of children and adults, as it is associated with both short- and long-term adverse life outcomes [1,2]. Studies have repeatedly shown the negative impact of child maltreatment on general and mental health [3-6] and quality of life [7-9].

The prevalence of child maltreatment in the Netherlands in the 0–18 years age range has been estimated at 34 per 1,000 children in 2010 [10]. In the Netherlands, suspected cases of child maltreatment can be reported to an advice and reporting center on child abuse and neglect (Advies- en Meldpunt Kindermishandeling or AMK), both by professionals and non-professionals, through a voluntary reporting system. The annual number of reports of suspected child maltreatment to these AMKs increased by 37.5% between 2006 and 2011 [11,12]. The AMKs investigate these reports and screen for problems in the family and its surroundings. If the report is substantiated, the AMK takes appropriate action, such as referring the child and family to child welfare and/or mental health organizations that can provide the necessary care. If voluntary care is rejected, the AMK can report the family to child protection services that can take court action or arrange the child to be placed in care outside the home. In case of substantiated violence, the AMK may file a report to the police [13].

Over the last decades the focus of the AMK investigations has changed from an adult-only approach to a more child-oriented approach using a Child-Interview intervention. In the adult-only intervention, only adults, including parents, teachers and general practitioners, are asked to provide information to substantiate the alleged abuse. Children are not used as informants in this intervention, for two reasons: the belief that children could not provide reliable and accurate information and the belief that children should not be troubled with questions about maltreatment. Regarding the first reason, several studies have shown that children can be reliable informants [14-17]. The Child-Interview intervention includes children aged 6 years and over as informants in the AMK investigation, in addition to adult informants. The children are interviewed by an AMK employee. Just as in the adult-only intervention, the aim of this interview is to collect information on the alleged child maltreatment, the existence of any family problems, the child’s needs for help and/or protection and the willingness to accept help and/or protection. An additional aim is to increase the child’s sense of being in control. AMK workers have confirmed the value and relevance of this information provided by children. In their experience, the maltreated children can provide highly relevant information that is otherwise not retrieved and this Child-Interview intervention therefore results in potentially better tailored interventions.

Regarding the second reason, several studies have shown positive effects on the children of talking about their maltreatment experiences. These studies showed that talking about such experiences may be the start of the recovery process and may increase children’s feelings of control and self-esteem [18-20], whereas keeping maltreatment experiences a secret is associated with additional trauma [21,22]. These findings suggest that the Child-Interview intervention is not only helpful for the referral to well-tailored care, but there are also benefits for the mental health and quality of life of these maltreated children. Nowadays all AMKs in the Netherlands have adopted the Child-Interview intervention and this is the preferred method according to the AMK manual [13]. Nevertheless, AMK records show that the adult-only intervention is still used in many AMK investigations. Little research has been done on the effects of specific AMK interventions [23], and scientific evidence on the presumed superiority of this Child-Interview intervention in comparison to an adult-only intervention is lacking.

In addition, it is well documented that children who have been abused or neglected are more likely to experience adverse outcomes in a number of domains throughout their lives. This results in an increasing demand on multiple services, including healthcare, by both children and their families [23]. Studies in the US have shown that substantial costs are associated with the impact of child maltreatment [24,25]. In the Netherlands, Meerding [26] estimated the economic burden of child maltreatment to be 965 million euros in 2003, which is about 138 euros per household annually. This was probably an underestimation, however, because the prevalence of child maltreatment in the Netherlands had not been studied yet and the estimates used were much lower than the recent prevalence rates reported by Alink and colleagues [10].

Due to scarcity of resources, the importance of economic evaluation studies has been increasingly acknowledged over the last decade. These studies provide information on the impact of child maltreatment on society and the costs of its treatment, which will help identify cost-effective resources. This knowledge contributes to informed decisions on the funding of services and to policy improvements [27,28]. So far, however, economic evidence on child maltreatment is scarce [27,29]. Corso and colleagues performed two literature reviews [27,29] and found that existing studies have focused mainly on cost of illness and do not include loss of quality of life. To our current knowledge, there have been five economic evaluations relating to child maltreatment, i.e. studies in which two or more interventions were analyzed in terms of the costs of effects. Three of these economic evaluations concerned prevention programs for parents [30-32]. The other two studies focused on sexually abused children in secondary care [33,34]. Economic evidence on the impact of child maltreatment and economic evaluations looking at interventions in this field have been scarce, especially in a Dutch context. The societal costs and effects of AMK involvement in the Netherlands have never been studied, even though AMKs have a pivotal function in stopping maltreatment, and thus in reducing its prevalence. Therefore, the primary aim of this study is to examine the effect of the participation of maltreated children aged 6–18 years in the AMK investigation (Child-Interview intervention) on their mental health and quality of life. As a second aim, this study will examine the balance between additional costs and effects of the Child-Interview intervention in comparison with AMK investigations without this intervention (adult-only intervention), from a societal perspective.

## Design/methods

### Study design

**Figure 1:**
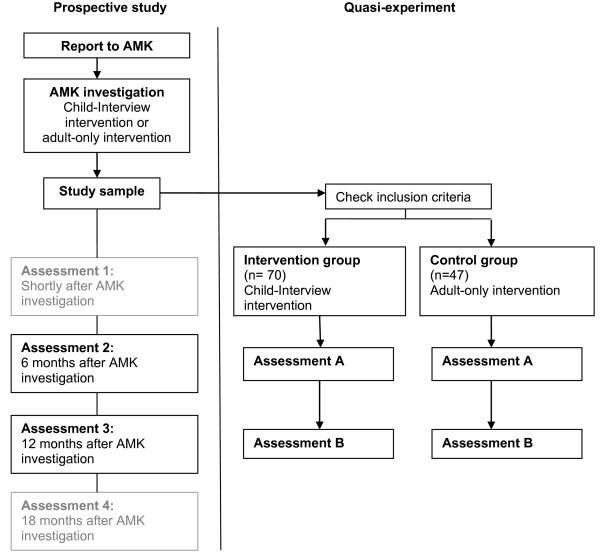
**Flow chart of quasi-experimental study design.***Note. Assessment 1 and 4 of the prospective study have no connection with the quasi-experiment.*

To answer the research questions, a quasi-experiment will be performed in which respondents from an ongoing prospective study on the mental health and quality of life of maltreated children after a report to an AMK [35] will be asked to complete an additional questionnaire at follow-up assessments. The quasi-experiment will consist of two post-intervention assessments of two nonequivalent groups (intervention group and control group) (see Figure 1). The study sample of the prospective study consists of maltreated children and their primary caretaker. These parent–child dyads were recruited for participation in the prospective study during the AMK investigation following a report to an AMK. Based on information from AMK records, the study sample will be divided into two groups: an intervention group, in which the Child-Interview intervention was used during the AMK investigation, and a control group, in which the intervention was not used (adult-only intervention).

The gold standard for experimental and effectiveness and cost-effectiveness studies is a randomized controlled trial (RCT), in which participants are divided to two study groups based on random assignment. The Dutch Medical Ethics Committee for Mental Health Care (METiGG) did not approve of a RCT, because randomization might result in a parent–child dyad receiving a suboptimal approach (in this case the adult-only intervention), which the committee decided was not ethically acceptable in the case of vulnerable young children. Although evidence on the adult-only intervention is lacking, this is supported by Article 33 of the Declaration of Helsinki. This article states that it is not acceptable to withhold treatment/intervention when it is known that this treatment can be lifesaving or that abstinence might lead to irrecoverable damage to the subject [36].

While preparing for the study, however, we found from AMK records that although the Child-Interview intervention is the preferred method according to the AMK manual [13], the Child-Interview intervention was not used during the investigation in a substantial number of AMK investigations. This enabled us to propose a quasi-experimental design in which an existing study sample (from the prospective study mentioned above) was divided into two groups based on the intervention they had received during the AMK investigation. Data collection for the quasi-experiment could be incorporated in this prospective study (see Figure 1). This design would make it possible to answer the research questions without randomization, though a disadvantage of this design is the possibility of selection bias. The economic evaluation will involve a combination of a cost-effectiveness analysis (CEA) and a cost-utility analysis (CUA).

### Assessment

This study will consist of two assessments: A and B (see Figure 1). Assessment A will take place at the same time as assessment 2 of the prospective study (6 months after the AMK investigation). Assessment B will take place six months later, at the same time as assessment 3 of the prospective study (12 months after the AMK investigation).

At assessments A and B, children and parents will be asked to complete a questionnaire. A member of the research team will meet the parent and child at a place of their choice (in most cases their own home) to assist them. The way in which the questionnaire is to be completed will be discussed with the parent and the child, and will depend on the participants’ reading and writing skills. If reading and writing skills are poor, a research team member will read out the questions and write down the participant’s answer on the questionnaire form. If reading and writing skills are sufficient, participants may choose to complete the questionnaire by themselves. To prevent the parents from influencing the children’s answers, parents and children will be requested to complete their questionnaires in separate rooms. Respondents will be rewarded for their participation by a 10 euro gift voucher for the parents, while the children will receive either a 5 euro gift voucher (children > 10) or an age-appropriate present (6–10 years).

### Target population

The target population will consist of maltreated children aged between 6 and 18 years and their primary caretaker. Inclusion criteria are (1) a report to an AMK about physical and/or emotional abuse, physical and/or emotional neglect and/or sexual abuse, (2) one child per family. When a report relates to more than one child of the same family, the oldest child within the age range will be included, (3) sufficient verbal and cognitive capacities of both parent and child, as the study will use mainly self-report methods for data collection, and (4) no intention to leave the Netherlands within the next 6 months, in view of the follow-up period.

### Sample size and recruitment

Participants from an ongoing prospective study on the mental health and quality of life of maltreated children after a report to an AMK [35] will be contacted to complete assessments A and B (see Figure 1). Parents and children will be asked to give written informed consent for their participation. Five dyads from the sample of this prospective study were excluded because they did not meet age criteria, while for 22 parent–child dyads maltreatment was not verified during the AMK investigation and for 17 dyads the type of maltreatment did not meet inclusion criteria of the quasi-experiment. Hence, the study sample for the present quasi-experiment will include 117 parent–child dyads. A sample size calculation confirmed that this is a sufficient number of participants. Based on the ability to detect a medium effect size or larger clinical effect (Cohen’s d > 0.45) [37] and tested at a conventional power of (1-beta) 0.80 and alpha of 0.05 (one-tailed testing), a total number of 110 parent–child dyads is required.

Supported by AMK records, this sample will be split up into two groups: an intervention group, in which the Child-Interview intervention was used during the AMK investigation, and a control group, in which the intervention was not used (adult-only intervention).

### Child-interview intervention vs. adult-only intervention in AMK investigation

All reports to an AMK are discussed in a multidisciplinary team meeting which draws up a plan for the investigation. It is at this team meeting that the choice of AMK investigation with or without the Child-Interview intervention is made, based on information from the person who reported the alleged maltreatment.

### AMK investigation without child-interview intervention

If the multidisciplinary team decides on AMK investigation without the intervention, only adults will be approached for information. The multidisciplinary team will decide which adults should be approached. Usually, the parents, teacher(s) and family doctor are part of this investigation, but other adults such as family members, social workers, therapists, or healthcare specialists can also be approached, depending on the social environment of the reported child/family. Information will be collected by phone, email contact or by a home visit.

### AMK investigation including the child-interview intervention

In addition to information from adults (see above), the child and his/her siblings will be asked as informants. The Child-Interview intervention comprises the child being interviewed by an AMK employee. This interview takes place at the child’s home or at school, in a separate room. The duration of the interview depends on the child’s needs. The aim of the interview is for the AMK employee to discover if there are problems in the family or its surroundings, if the child needs care and what type of care would be suitable. An additional aim is to increase the child’s sense of being in control.

The Child-interview intervention used by the AMKs is part of the investigation to substantiate a report of suspected child maltreatment and to identify the needs for child and family care. It does not serve a forensic purpose.

### Outcome measures

#### (1) Effectiveness study

##### Mental health

Children’s mental health (in terms of internalizing and externalizing psychological problems) as observed by the parents, will be measured with the Dutch version of the Child Behavior Checklist (CBCL) [38]. Parents will be asked to what extent they observe various behavioral and emotional problems in their child. The CBCL uses a 3-point scale and consists of 113 items. Internal consistency is good (α .97) [38].

##### Quality of life

Children’s quality of life will be measured with one of three age-appropriate versions (5–7, 8–11, 12–18 years) of the Dutch translation of the Pediatric Quality of Life Inventory (PedsQL) [39]. Children will be asked to express their concerns on the dimensions of physical health and psychosocial health, the latter consisting of the subdimensions of emotional functioning, social functioning and school functioning. The overall quality of life score will be obtained by adding up the scores on all dimensions. The PedsQL uses a 5-point scale (or a 3-point scale for the 5–7 version) and consists of 23 items. Internal consistency is good (α .82- .85) [39].

#### (2) Economic evaluation study

##### Societal costs

Healthcare costs of the child and the family will be measured using an adapted version of the Trimbos Institute Medical Technology Assessment Cost Questionnaire for Psychiatry (TiC-P) [40]. This questionnaire will be completed by the parent. As the TiC-P is not completely suitable for this intervention, the TiC-P had to be adapted, using a bottom-up approach. In this procedure, AMK managers and AMK workers were asked to review the TiC-P for the purpose of studying a maltreated population and they were asked to add services that families that are reported to an AMK use regularly and that were missing from the TiC-P. This resulted in a suitable questionnaire to record societal costs of maltreated children and their families. The updated version of the TiC-P questionnaire included the following services: general practitioner, company doctor, first aid post at hospital, medical specialist at hospital, hospitalization (including general hospital, psychiatric hospital, rehabilitation center, university hospital), psychiatrist/psychologist/psychotherapist working at hospital, psychiatrist/psychologist/psychotherapist with private practice, physical therapist, alternative healer (e.g. homeopathy), self-help group therapy, child welfare service on outpatient basis, child welfare service as day care, child welfare service on inpatient basis, AMK, youth and family center, infant welfare center, regional public health service, home care, personal care budget (allocated by local government), social worker, school (including school doctor, compulsory education officer), police, probation service, addiction service, financial assistance, and lawyer/legal aid.

Costs will be valued on the basis of guideline prices derived from the updated Dutch manual for cost analysis in healthcare research [41]. As the guideline only includes healthcare costs, costs outside the healthcare sector will be valued separately.

### Quality adjusted life years (QALYs)

An increased quality of life is expressed as a *utility* value on a scale from 0 (death) to 1 (perfect quality of life). In health economics, utilities are combined with survival estimates and aggregated across individuals to generate quality adjusted life years (QALYs). A one-year increase in the duration of life (without change in quality of life), or an increase in quality of life from 0.5 to 0.7 utility units for five years, would both result in a gain of one QALY. Children’s QALYs will be derived from a thermometer question. Children will be asked to answer the question: “*On a scale from 1 to 10, how healthy did you feel over the past 6 months?”* The scores will be converted to utility scores by dividing by 10, obtaining a score between 0.00 and 1.00. The change in utility value between the two assessment points will be multiplied by the duration of the intervention effect to obtain the number of QALYs gained.

### Analyses

Data will be analyzed according to the intention-to-treat principle. The similarity of baseline characteristics (socio-demographic characteristics, costs and outcomes) between the two AMK investigation interventions will be examined using univariate tests and Chi-square tests. Loss-to-follow up will be accounted for by imputing missing data using regression imputation.

#### (1) Effectiveness study

A multivariate regression analysis will be performed in which outcomes of mental health and quality of life will be compared between the Child-Interview intervention group and the control group (adult-only intervention). This will be done at follow-up (assessment B), 6 months after assessment A and a year after the intervention. Covariates will be taken into account.

#### (2) Economic evaluation study

In this study, additional costs and additional outcomes of the Child-Interview intervention will be compared with those of the adult-only intervention. The incremental cost-effectiveness ratio (ICER) will be expressed as the incremental costs per degree of improvement in mental health of the Child-Interview intervention group in comparison with the control group (adult-only intervention). The incremental cost-utility ratio (ICUR) will be expressed as the incremental costs per QALY of the Child-Interview intervention group in comparison with the control group (adult-only intervention).

The ICER (and ICUR) will be calculated using ICER = (Ci-Cc)/(Ei-Ec), where Ci is the annual total costs of the Child-Interview intervention, Cc the annual costs of the adults-only intervention, Ei the effect at 6-months follow-up for the Child-Interview intervention and Ec the effect at 6-months follow-up of the adult-only intervention. The robustness of the ICER will be checked by non-parametric bootstrapping (1000 times). The bootstrap replications will be used to calculate 95% confidence intervals around the costs. The bootstrapped ratios (ICER and ICUR) will be graphically presented in two ways: (1) plotted in a cost-effectiveness plane, in which the vertical line reflects the difference in costs and the horizontal line reflects the difference in effects, and (2) in a cost-effectiveness acceptability curve, showing the probability that the Child-Interview intervention is cost-effective based on a range of ceiling ratios (the maximum amount of money society is willing to pay to gain one extra unit of effect or a gain in QALY). Additionally, the robustness of the base-case findings will be assessed using a sensitivity analysis [42]. This economic evaluation will be performed from a societal perspective. Bootstrapping will be carried out in Excel.

### Collaboration

This study is a joint project of Trimbos institute, the Netherlands Institute for Mental Health and Addiction (Utrecht), the CAPHRI School for Public Health and Primary Care, Maastricht University and VU University Amsterdam. The research is funded by the Netherlands Organisation for Health Research and Development (ZonMw) (project No. 15700.2012) and is registered in the Netherlands Trial Register, part of the Dutch Cochrane Centre (NTR3728). This study was approved by the Dutch Medical Ethics Committee for Mental Health Care (METiGG) in February 2012 (NL31267.097.10).

## Discussion

The value of having children participate in the AMK investigations by using the Child-Interview intervention is increasingly being recognized. The intervention yields valuable information to substantiate the alleged maltreatment report, and the intervention is also expected to be beneficial for the mental health and quality of life of the maltreated children. However, this beneficial effect has not yet been studied. In addition, child maltreatment is associated with substantial societal costs because of the high demand for healthcare and other services by both the maltreated children and their families. Using the Child-Interview intervention may prevent or reduce negative outcomes of child maltreatment, which may result in a lower consumption of healthcare and other services.

Although the AMK manual suggests that using the Child-Interview intervention during AMK investigation is the preferred method, the AMKs in the Netherlands are still in a transition period as regards the nature of the investigation. From a small survey among AMKs we estimate that the adult-only intervention is still used in approximately 40% of all investigations by the AMKs. At this point in time, this enables us to answer the research questions by means of the proposed quasi-experimental design, before the Child-Interview intervention is fully adopted, as the percentage of cases in which the intervention is not used is still sufficient. We will be able to derive two study groups with acceptable sample sizes from an ongoing study examining the mental health and quality of life of children reported as being maltreated.

A strong point of the proposed study is the use of child self-reports to collect data on quality of life, one of the main outcome measures of the effectiveness study. Data on quality of life are often collected using proxies, but research findings show discrepancies between child and proxy reports [43]. As a consequence, the value of obtaining children’s self-reports is increasingly recognized and several studies have established the accuracy and reliability of child reports [39,43-45].

Furthermore, not only will this be the first study to examine the effect of AMK involvement, it will also be the first economic evaluation performed in the Netherlands regarding an intervention for maltreated children. Since economic evaluations on child maltreatment have been scarce, it is expected that not all costs of healthcare and other services will be available yet. These costs will need to be valued for this study, so some of the costs will be based on estimates.

Additionally, many aspects of utility measurement in children have not yet been fully developed. There is a lack of health state classification instruments that consider children’s developmental stages, and there is a need to understand the role of proxies in assessing and valuing children’s health [46]. This is why we chose an alternative method, a visual analogue scale. The advantage of the thermometer method that will be used is that it is simple and therefore easily understood by children. However there are also disadvantages to this method, in that it generates values rather than utilities and does not involve an element of choice, nor decision-making under uncertainty, which may result in lower utility estimates.

A methodological concern for the quasi-experimental design of this study is the potential selection bias. All reports that are filed with the AMKs are discussed in a multidisciplinary team in which decisions about the best AMK investigation approach (Child-Interview intervention vs. adult-only intervention) are made. The choice of intervention is based on information collected from the reporter of the suspected child maltreatment. This reporter can be either a professional or non-professional (such as a neighbor), which may affect the quality and reliability of the information on which the multidisciplinary team must base their decision. In addition, AMK investigation using the Child-Interview intervention is the preferred method according to the manual for AMK investigations [13]. When the adult-only intervention is nevertheless chosen, there may be specific reasons for this, such as parents refusing their child’s participation in the AMK investigation, the child having been seen by a therapist or social worker, in which case this professional is approached to prevent additional strain upon the child, and/or language barriers. As a result of these limitations, the two groups may not be comparable at baseline, causing a concern regarding internal validity.

Another limitation is the possible recall bias caused by the time that elapses between the assessment moments. A period of 3 months is generally used between assessments in economic evaluations. However, this study will use a period of 6 months because the assessment moments will be connected to those of the ongoing prospective study. Respondents may have difficulty remembering the exact number of times they used healthcare and other services, which may affect outcomes.

To conclude, within the constraints of ethical and legal standards, the quasi-experimental study design we chose is expected to provide information on the optimal approach for AMK investigation, in terms of effectiveness and cost-effectiveness. The aim of an AMK investigation is to refer the child and family to well-tailored care and to end substantiated maltreatment. The use of the Child-Interview intervention may contribute to the prevention of negative outcomes of child maltreatment, if using this intervention during the AMK investigation proves to result in better mental health and quality of life outcomes for maltreated children because well-tailored care can be provided on the basis of information that cannot be retrieved with the adult-only intervention. Policy makers are increasingly interested in the cost-effectiveness of methods being used, and cost-effectiveness studies are increasingly used in the development of new policies.

## Competing interests

All authors declare that they have no competing interests.

## Authors’ contributions

All authors participated in describing the design of this study. FS drafted the manuscript. CH, FLW, PB and SMAAE contributed to the acquisition of funding for this study and participated in the writing of the manuscript by critically revising draft versions. All authors have read and approved the final manuscript.

## Pre-publication history

The pre-publication history for this paper can be accessed here:

http://www.biomedcentral.com/1471-2458/13/1164/prepub
